# Variation in vitamin D supplementation among adults in a multi-race/ethnic health plan population, 2008

**DOI:** 10.1186/1475-2891-11-104

**Published:** 2012-12-11

**Authors:** Nancy P Gordon, Bette J Caan, Maryam M Asgari

**Affiliations:** 1 Kaiser Permanente Northern California’s Division of Research in Oakland, Oakland, CA, USA

**Keywords:** Vitamin D supplementation, Multivitamin supplementation, calcium supplementation, differences in vitamin D supplementation, gender differences in vitamin D supplementation

## Abstract

**Background:**

Vitamin D may have a role in many chronic conditions in addition to bone health. Nutritional surveys among Americans have reported high levels of vitamin D insufficiency, especially among Blacks and Latinos. Our study examined variation in vitamin D supplementation practices in an adult health plan population by age, gender, and race-ethnicity.

**Methods:**

Self-report data from a 2008 general health survey in a large Northern California health plan were used to characterize number and types of sources of vitamin D supplementation (multivitamin, calcium with D, singular D) among women and men aged 25-85, overall, by race-ethnicity, and for obese, diabetic, and hypertensive subgroups.

**Results:**

In this population, 40% of women and 54% of men ≤ 50, and 24% of women and 53% of men aged 51-85 get no vitamin D from dietary supplements. Higher vitamin D supplementation among women > 50 is associated with higher reported intake of calcium with D. Black and Latina women aged 25-85 and Filipinas in the ≤ 50 age group were significantly less likely than non-Hispanic Whites to get vitamin D from supplements, whereas vitamin D supplementation practices among Chinese women did not significantly differ from non-Hispanic Whites. Among men, Latinos aged 25-85 and Black and Chinese ≤ 50 were significantly less likely than non-Hispanic Whites to get vitamin D from supplements. Similar race-ethnic differences in vitamin D supplementation patterns were observed for people in the obese, diabetic, and hypertensive groups.

**Conclusions:**

Our survey results suggest that in 2008, a large percentage of women and an even larger percentage of men in a large Northern California health plan get no vitamin D from dietary supplements, and that Blacks and Latinos and obese adults, who are at higher risk of vitamin D deficiency, are also the least likely to get any vitamin D from dietary supplements.

## Background

It is well-accepted that low vitamin D can cause bones to become brittle and misshapen (rickets) [1]. Further, clinical trials have shown that vitamin D supplementation can reduce osteoporosis, decrease risk of falls, and impact all-cause mortality among healthy middle aged and elderly adults [2,3]. Less conclusive and less well studied is the relationship of low vitamin D to increased risk of a growing number of chronic illnesses (various cancers, diabetes, hypertension, heart disease, kidney disease, asthma, and autoimmune diseases), infectious respiratory diseases, pregnancy-related problems, and adverse birth outcomes seen in observational studies [4-29]. Recent clinical research has shown that most tissues and cells in the human body have a vitamin D receptor, and that several possess the ability to convert the primary circulating form of vitamin D, 25-hydroxyvitamin D, to the active form, 1,25-dihydroxyvitamin D [30]. At the genetic level, researchers have also found over 2,700 binding sites for the vitamin D receptor along the length of the human genome, with unusually large concentrations near a number of genes associated with autoimmune conditions, certain cancers, and type 1 diabetes. However, in 2010, an Institute of Medicine (IOM) expert panel reviewed the evidence for adequate intake of vitamin D and calcium and found no strong cause-and-effect evidence that remediating vitamin D insufficiency would prevent the development of any conditions other than osteoporosis [31].

There is evidence that a substantial percentage of the U.S. population has serum vitamin D concentrations below the levels currently recommended for people their age [32,33]. Based on cut-points that define serum 25-hydroxyvitamin D (25[OH]D) as deficient if <20 ng/mL and insufficient if 21–29 ng/mL [6], NHANES 2001–2004 showed that 77% of the U.S. population was vitamin D insufficient, a significantly higher prevalence than was seen in the 1994–1998 NHANES [34]. However, there were striking race-ethnic differences. While approximately 70% of adult nonHispanic white men and women were vitamin D insufficient, over 95% of nonHispanic Black/African-American men and women and approximately 90% of Latino men and women had insufficient vitamin D concentrations [34]. Another study using NHANES 2004–2006 serum data found that approximately 42% of U.S. adults were vitamin D deficient, with significantly higher prevalence of deficiency among Blacks (82%) and Latinos (62%) compared with nonHispanic Whites (30%) [33]. Studies of well-characterized populations have also found very high levels of vitamin D insufficiency and deficiency in the U.S. population, including Black and Latino adolescents and adults [35-37], pregnant women [38,39], older adults [40], obese (BMI ≥ 30) adults [33], hypertensives [33], and people with low HDL cholesterol [33]. While the IOM and Endocrine Society recently reaffirmed the cut-point for vitamin D deficiency as <20 ng/mL [41,42], many researchers have also started to question whether this is too low, suggesting that < 32 ng/mL is a more appropriate cut-point based on an array of biomarkers that have been show to be adversely affected by vitamin D deficiency [43,44]. This would obviously make the prevalence of vitamin D deficiency and insufficiency in the U.S. population, and especially in high risk groups, much higher.

Based on a review of evidence, in 2010, the IOM expert panel on vitamin D increased the recommended daily allowance (RDA) for vitamin D to 600 IU/day for children and adults through age 70 and 800 IU/day for ages 71 and older [41]. Prior to this, the RDA was 200 IU/day for children and adults through age 50, 400 IU/day for adults aged 51–70, and 600 IU/day for adults ages 71 and older [41].

Surveillance studies suggest that only a small percentage of the U.S. population gets adequate vitamin D from food sources. According to NHANES 2005–2006, only 10% of adults aged 51–70 and 15% of those aged ≥71 met the AI (average daily intake) for vitamin D from food based on 1997 dietary reference intake (DRI) criteria [45]. Further, data from the 1988–1994 and 1999–2000 NHANES show that starting about the time of puberty, usual vitamin D intake from food of African-Americans is significantly below that of nonHispanic Whites and Latinos in every age group, with the exception that DAI of Latinos drops to the level of African-Americans in the ≥71 age group [46,47].

Before 2010, when singular D supplements started to become more widely available, vitamin D was primarily obtained from daily multivitamins and calcium plus D. According to NHANES data for 2003–2006, 56% of women aged ≥60, 45% of women aged 40–59, and 33% of women aged 20–39 got vitamin D from one or more dietary supplements, up significantly from 1999–2002. Vitamin D supplementation among men was significantly lower than that of women in the same age groups (44%, 38%, and 26%, respectively) and had not changed from the earlier time period [46]. As with vitamin D intake from food, NHANES data indicated that African-American and Latina women were significantly less likely to take calcium and multivitamin supplements than nonHispanic White women [48-52].

In order to characterize vitamin D supplementation practices among insured adults in Northern California and learn whether supplementation practices differ by race-ethnicity, we analyzed data obtained from the 2008 Kaiser Permanente Northern California (KPNC) adult Member Health Survey. Our study had two main objectives: (1) to describe vitamin D supplementation practices of adult men and women in different age groups and with metabolic conditions that may increase risk (obesity) or be affected by (diabetes, hypertension) vitamin D deficiency; and (2) to determine whether vitamin D supplementation practices vary by race/ethnicity. A secondary objective was to investigate whether sources of vitamin D from supplements varied by age, gender, and race/ethnicity.

## Methods

### Data source

Data for this study come from the 2008 Kaiser Permanente Northern California (KPNC) Member Health Surveys (MHS) [53]. This survey is conducted every three years using a confidential self-administered questionnaire (hardcopy or link to an online version) sent to an age-, gender-, and geographically stratified random sample of KPNC adult members. The KPNC adult population is highly comparable to the population of insured adults in Northern California with regard to demographic and health-related characteristics, with the exception of a smaller percentage of adults at very low levels of income and education [54]. The MHS collects information about a variety of member characteristics including demographics, selected health conditions and overall health status, and health-related behaviors, including use of dietary supplements. The survey is funded by KPNC’s Community Benefit Program and approved by KPNC’s Institutional Review Board.

### Study variables

The MHS dietary supplement question asked “During the *past 12 months*, did you use any herbals, nutritional supplements, or other “natural” remedies *to treat or prevent your own health problems*?” Daily multivitamin, calcium with D, and calcium without D (including Tums or Rolaids) were included as checklist items, and respondents were also asked to write in additional dietary supplements which were subsequently coded (this latter was the source of vitamin D alone or as part of another non-calcium, non-multivitamin supplement). In 2008, a regular adult multivitamin, calcium with D tablet (calciumD), and singular vitamin D tablet each usually provided 400 IU of vitamin D_2_. Based on labeled recommended dosages, intake of more than one of these supplements daily would be needed for an individual to get over 400 IU of vitamin D from dietary supplements. Age, gender, race/ethnicity, education, obesity (BMI ≥ 30), non-gestational diabetes, and hypertension were also assigned based on self-reported survey data.

The study sample consisted of all respondents aged 25–85 who provided information about their use of multivitamin and calcium supplements. There were 8,884 women (3,531 aged 25–50, 3,116 aged 51–70, and 2,237 aged 71–85) and 7,165 men (2,119 aged 25–50, 2,737 aged 51–70, and 2,308 aged 71–85). The numbers of respondents in the five race/ethnic groups and three health risk groups can be found in Table 1. Subgroup comparisons were restricted to these five race/ethnic groups because of small numbers in other ethnic groups.

**Table 1:** Characteristics of Study Sample

	**Women Ages 25–50 yr**						**Women ages 51–85 yr**					
	**All**	**WhiteNH**	**Black**	**Latina**	**Filipina**	**Chinese**	**All**	**WhiteNH**	**Black**	**Latino**	**Filipino**	**Chinese**
	**(N=3531)**	**(N=1830)**	**(N=263)**	**(N=541)**	**(N=282)**	**(N=212)**	**(N=5353)**	**(N=3806)**	**(N=376)**	**(N=389)**	**(N=271)**	**(N=192)**
	**Wtd. %**	**Wtd. %**	**Wtd. %**	**Wtd. %**	**Wtd. %**	**Wtd. %**	**Wtd. %**	**Wtd. %**	**Wtd. %**	**Wtd. %**	**Wtd.%**	**Wtd. %**
**Age (mean yrs)**	37.8	38.5	37.9	37.4*	37.3	37.0	62.7	63.4	61.6*	60.9*	60.3*	60.9*
**Race/Ethnicity**												
White nonHispanic	52.5	–	–	–	–	–	70.3	–	–	–	–	–
Black	7.0	–	–	–	–	–	6.4	–	–	–	–	–
Hispanic/Latina	15.5	–	–	–	–	–	7.4	–	–	–	–	–
Filipina	7.6	–	–	–	–	–	5.4	–	–	–	–	–
Chinese	5.6	–	–	–	–	–	3.8	–	–	–	–	–
Other Asian/Pac Isl.	9.0	–	–	–	–	–	4.6	–	–	–	–	–
Other	2.8	–	–	–	–	–	2.1	–	–	–	–	–
**Education**												
≤High School Graduate	13.2	11.1	17.4	24.8*	7.0	4.8	25.1	23.7	26.6	45.6*	17.9	18.1
Some College	36.5	36.3	48.0	43.8	32.5	15.7	38.4	39.4	51.8	35.6	21.8	36.7
College Graduate	50.3	52.6	34.6*	31.4*	60.5	79.5	36.5	36.9	21.6*	18.8*	60.2	45.2
**Income (2007)**												
< $35,000	13.8	12.1	23.1*	16.6	14.0	6.9*	26.0	23.9	40.6*	37.1*	33.8*	14.1
$35,000-$50,000	14.0	11.2	20.9	21.1	16.1	11.5	14.8	14.9	16.9	14.3	12.4	14.9
50,001-$80,000	26.0	26.4	32.0	27.0	25.0	20.4	24.8	25.3	21.9	24.9	21.8	26.2
$80,001-$100,000	14.1	14.9	5.6	15.3	15.5	9.0	12.2	12.3	10.2	11.1	13.8	15.1
> $100,000	32.1	35.4	18.4*	20.0*	29.4	52.2*	22.2	23.6	10.4*	12.6*	18.2	29.7
**Health Status**												
Good or better health	92.8	93.8	88.1*	89.7*	93.6	96.3	85.1	86.5	77.4*	78.2*	83.0	90.8
Diabetic	4.1	3.5	5.9*	5.2	9.1	2.0	12.9	10.5	24.0*	20.1*	20.9*	9.4
Hypertensive	10.2	9.8	16.3*	8.6	17.7*	6.8	46.4	44.8	64.8*	45.4	57.4*	38.0
Obese (BMI ≥ 30)	25.8	26.0	41.3*	34.9*	16.8*	7.2*	27.7	28.0	47.0*	39.3*	8.9*	9.1*
**Age (mean yrs)**	37.8	38.5	37.9	37.4*	37.2	37.0	62.7	63.4	61.6*	60.9*	60.3*	62.7
**Race/Ethnicity**												
White nonHispanic	53.0	–	–	–	–	–	70.1	–	–	–	–	–
Black	5.7	–	–	–	–	–	5.2					
Hispanic/Latino	15.6	–	–	–	–	–	8.2					
Filipino	7.3	–	–	–	–	–	5.5					
Chinese	6.0	–	–	–	–	–	3.6					
Other Asian/Pac. Isl.	9.9	–	–	–	–	–	5.1					
Other	2.5	–	–	–	–	–	2.3					
**Education**												
≤High School Graduate	17.9	16.9	23.7	31.9*	13.9	3.7	21.2	19.9	23.7	35.0*	16.0	16.2
Some College	34.4	35.2	46.0	37.7	34.3	17.2	36.3	36.8	44.7	38.0	35.7	19.6
College Graduate	47.7	47.9	30.3*	30.4*	51.8	79.1*	42.5	43.3	31.6*	27.0*	48.3	64.2
**Income (2007)**												
< $35,000	10.4	9.0	17.0	11.3	10.3	3.9	17.3	15.0	22.4	24.3*	25.2*	13.7
$35,000-$50,000	12.6	10.3	15.5	22.1	13.3	5.0	13.6	13.2	14.6	11.8	14.5	19.6
$50,001-$80,000	27.2	26.5	32.3	30.6	33.6	26.6	24.4	24.3	24.2	30.4	22.9	17.5
$80,000-$100,000	14.5	15.0	11.3	13.1	12.3	16.3	13.3	14.2	12.6	10.5	14.5	10.2
> $100,000	35.3	39.2	23.9*	22.9*	30.5	48.2	31.4	33.3	26.2	23.0*	22.9*	39.0
**Health Status**												
Good or better health	93.7	94.4	89.1*	93.3	93.0	96.5	83.7	83.9	79.8	82.0	85.7	87.4
Diabetic	4.3	2.9	10.6*	5.3	6.2	2.3	17.8	15.2	25.5*	26.8*	31.0*	16.2
Hypertensive	13.2	12.1	20.4	13.2	22.0*	7.8	49.9	48.3	64.2*	51.1	61.4	41.3
Obese (BMI ≥ 30)	25.1	25.7	34.5	34.2	18.2	9.8	26.4	27.3	31.1	39.3*	17.2*	2.8*

Wtd. %=Respondent data weighted to the age, gender, and geographic composition of the health plan membership at time of the survey. WhiteNH=nonHispanic White; Pac. Isl, = Pacific Islander.

* Differs significantly from nonHispanic Whites at p<.05.

### Statistical analysis

Because supplementation practices are known to differ significantly by gender, we conducted all analyses separately for men and women. To study differences by age, we first analyzed vitamin D supplementation practices separately for three age groups (25–50, 51–70, and 71–85). Because we found no significant differences in supplementation patterns between the 51–70 and 71–85 age groups, these age groups were combined for race-ethnic and health risk groups analyses. Race-ethnicity analyses were restricted to five groups (nonHispanic White, African-American/Black, Latino/a, Filipino/a, and Chinese) due to sample size, and in the case of obese and diabetic patients, comparisons omit the Asian groups due to small numbers.

**Figure 1:**
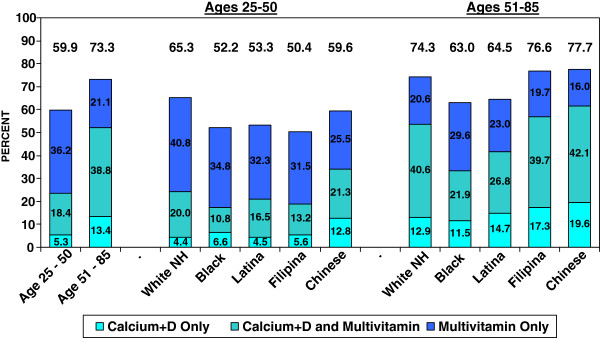
**Percentages of Women Who Got Vitamin D from a Calcium+D and/or a Daily Multivitamin Supplement, by Age and Race/Ethnicity, 2008.** Note: Less than .2% of 25–51 year olds and less than .5% of 51–85 year olds got vitamin D supplementation from another source in 2008.

**Figure 2:**
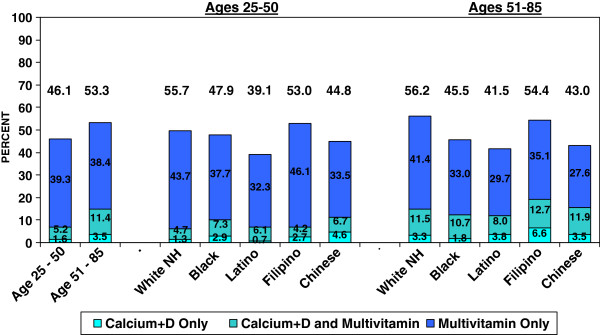
**Percentages of Men Who Got Vitamin D from a Calcium+D and/or a Daily Multivitamin Supplement, by Age and Race/Ethnicity, 2008.** Note: Less than 0.2% of 25–51 year olds and less than 0.5% of 51–85 year olds got vitamin D supplementation from another source in 2008.

All analyses used respondent data weighted using a post-stratification weighting factor that made the final respondent sample reflect the actual age (by 5-year intervals), gender and geographic distribution of the adult membership from which the sample was drawn. While percentages reported in the text and tables are based on weighted data, the table Ns are actual unweighted subgroup denominators used in the analyses. All percentages were calculated using the Proc Surveymeans procedure in PC-SAS version 9.2 for data collected using a complex survey design [55], and significance was assessed based on overlap of the 95% confidence intervals (CI) around the percentages. To assess whether daily multivitamin, calciumD, and vitamin D supplementation (none, from one source, or from ≥ 2 sources) patterns differed by race/ethnicity after adjusting for age and educational attainment, we ran gender-specific logistic regression models (Proc Surveylogistic) with indicator variables to represent African-American/Black (Black), Latino/a, Filipino/a, and Chinese compared against nonHispanic Whites (WhiteNH) and continuous variables for age (5 year intervals) and education (≤ high school graduate, some college, college graduate). Confidence intervals for percentages in Tables 1, 2, 3 and Figures 1,2 and the adjusted odds ratios and CIs from the logistic regression models that are not presented in the paper are available upon request.

**Table 2:** Percentages of adults taking a multivitamin, calcium, and getting vitamin D from supplements in 2008 by age group, gender and race/ethnicity

				**Vitamin D from a Supplement**^**1**^			
		**Daily Multivitamin**	**Calcium+D Supplement**	**None**	**From 1 source**	**From ≥ 2 sources**	
	**Unwtd. N**	**Wtd %**	**Wtd. %**	**Wtd. %**	**Wtd. %**	**Wtd. %**	
**WOMEN**							
25-50 yr	3531	54.6	23.7	40.0	41.6	18.4	
51-70 yr	3116	60.2	51.3	26.9	34.3	38.8	
71-85 yr	2237	58.4	54.3	25.7	35.5	38.8	
**Age 25-50**							
WhiteNH	1832	60.8	24.4	34.7	45.3	20.0	
Black	263	45.6*	17.4	47.8*	41.4	10.8*	
Latina	541	48.7*	21.0	46.7*	36.8	16.5	
Filipina	282	44.7*	18.9	49.6*	37.2	13.2*	
Chinese	212	46.8*	34.1*	40.4	38.3	21.3	
**Age 51-85**							
WhiteNH	3806	61.2	53.3	25.7	33.7	40.6	
Black	376	51.2*	33.4*	37.0*	41.1	21.9*	
Latina	389	49.8*	41.4*	35.5*	37.7	26.8*	
Filipina	271	59.4	57.0	23.3	37.0	39.7	
Chinese	192	58.1	61.5	22.3	35.6	42.1	
**MEN**							
25-50 yr	2120	44.5	6.8	53.8	40.9	5.2	
51-70 yr	2737	49.5	13.4	47.3	42.3	10.4	
71-85 yr	2308	50.8	19.7	44.2	40.8	15.0	
**Age 25-50**							
WhiteNH	1127	48.4	6.1	50.1	45.1	4.8	
Black	119	45.0	9.6	52.1	40.6	7.3	
Latino	324	38.4*	6.8	60.9*	33.0	6.1	
Filipino	153	50.3	6.9	47.0	48.8	4.2	
Chinese	128	40.2	11.3	55.2	38.1	6.7	
**Age 51-85**							
WhiteNH	3646	52.9	14.7	43.8	45.2	11.4	
Black	285	43.7	12.2	54.2*	35.1	10.7	
Latino	371	37.8*	11.8	58.5*	56.5	8.0	
Filipino	233	47.8	17.9	45.7	41.6	12.7	
Chinese	186	39.4*	15.3	57.1*	31.0	11.9	

Unwtd. N = Actual subgroup sample size. Wtd. % = Respondent data weighted to the age, gender, and geographic composition of the health plan membership at time of the survey.

^**1**^ Sources of vitamin D: Calcium with D, multivitamin, singular D, or D in another combination. At the time of the survey, each of these sources generally contained 400 IU of vitamin D_2_.

* Differs significantly from White nonHispanics at p<.05.

**Table 3:** Vitamin D supplementation by men and women aged 25–85 in selected health risk groups

		**Number of Vitamin D Sources**^**1**^			
	**Unwtd**	**None**	**1 Source**	**≥ 2 Sources**	
	**N**	**Wtd. %**	**Wtd. %**	**Wtd. %**	
**Obese(BMI ≥30)**^**2**^					
**Women**					
All	2324	40.2	36.8	23.0	
WhiteNH	1463	34.4	38.9	26.7	
Black	290	47.4*	39.4	12.9*	
Latino	333	55.0*	29.9	15.1*	
**Men**					
All	1707	51.2	41.5	7.3	
WhiteNH	1164	47.9	44.9	7.1	
Black	131	49.0	41.4	9.5	
Latino	234	60.2*	32.7	7.1	
**Diabetic**^**2**^					
**Women**					
All	885	37.3	36.2	26.5	
WhiteNH	490	30.9	38.5	30.5	
Black	108	48.0*	41.6	12.4*	
Latino	121	54.1*	27.5	18.4*	
**Men**					
All	1125	49.4	41.2	9.4	
WhiteNH	685	45.3	44.0	10.6	
Black	96	50.4	36.1	13.5	
Latino	129	60.3*	37.5	2.2*	
**Hypertensive**					
**Women**					
All	3169	33.0	36.6	32.0	
WhiteNH	2094	28.8	35.8	35.3	
Black	316	39.4*	44.0	16.6*	
Latino	252	41.5*	34.5	24.0*	
Filipino	225	39.7*	35.5	24.8*	
Chinese	101	29.5	32.7	37.7	
**Men**					
All	3091	46.7	43.4	9.9	
WhiteNH	2099	43.9	46.0	10.1	
Black	241	47.1	44.3	8.6	
Latino	246	56.3*	33.2	10.5	
Filipino	183	46.0	44.2	9.8	
Chinese	112	52.5	37.6	9.9	

Unwtd. N = Actual subgroup sample size. Wtd. % = Respondent data weighted to the age, gender, and geographic composition of the health plan membership at time of the survey.

^1^ Sources of vitamin D: Calcium with D, multivitamin, singular D, or D in another combination. At the time of the survey, each of these sources generally contained contained 400 IU of vitamin D_2_.

^2^ Numbers of Filipino and Chinese women and men in risk group too small to use for estimates.

* Differs significantly from White nonHispanics at p<.05.

## Results

Table 1 presents characteristics of the women and men by age group and race-ethnicity within age group. The average ages of the younger and older groups were 37.7 yrs and 63 yrs, respectively. Within each age group, the average age of WhiteNH and Black women and men tended to be higher than that for the Latino/as, Filipino/as, and Chinese, but most of the differences were not statistically significant. Blacks and Latinos were significantly less likely than nonHispanic Whites to be college graduates and to have household incomes > $100,000. Among women, Blacks and Latinas were significantly less likely than WhiteNH, Filipinas, and Chinese to report being in good health and significantly more likely to be obese. Black women in both the younger and older age groups had a significantly higher prevalence of diabetes and hypertension than WhiteNH women, and among women aged > 50, Latinas and Filipinas also had a higher prevalence of diabetes. While differences in health-related characteristics among men in the five race-ethic groups were very similar to those among the women, due to smaller sample sizes, fewer comparisons achieved statistical significance.

The estimated prevalence of vitamin D supplementation by gender, age group, and race/ethnicity is shown in Figure 1 and Table 1. Among 25–50 year olds, 40% (CI: 38.3%-41.8%) of women and 54% (CI: 51.4%-56.2%) of men were getting no vitamin D from dietary supplements and only 18% (CI: 17.0-19.8%) and 5% (CI: 4.2-6.2%), respectively, were getting vitamin D from ≥ 2 sources. Among 51–85 year olds, approximately 27% (CI: 25.2-28.1%) of women and 46% (CI: 44.8-48.4%) of men were getting no vitamin D from dietary supplements, while only around 39% (CI: 37.2-40.3%) of women and 11% (CI: 10.3-12.5%) of men were getting it from ≥ 2 sources. As this figure also shows, the age and gender-related differences in vitamin D supplementation were primarily due to significantly greater use of calciumD by women over age 50.

As indicated in Figure 1- 2 and Table 2, we also found that within age groups, there were race/ethnic differences in vitamin D supplementation practices. Across both age groups, Black and Latina women were significantly less likely than nonHispanic Whites to be getting any vitamin D from dietary supplements, and in the age 25–50 group, Filipinas were also significantly less likely than nonHispanic Whites to be getting any vitamin D from supplements. In both age groups, Black women were half as likely as nonHispanic White women to be getting vitamin D from ≥ 2 sources. Filipinas in the younger age group and Latinas in the older age group were also significantly less likely than nonHispanic White women to be getting vitamin D from ≥ 2 sources. The prevalence of vitamin D supplementation practices among Chinese women did not significantly differ from that of nonHispanic White women except for the percentages of women getting their vitamin D from calciumD. Among men, in both age groups Latinos were significantly less likely than nonHispanic Whites to be getting any vitamin D from supplements, with Black and Chinese men also less likely to be getting vitamin D from supplements among those over the age of 50. There were no significant race-ethnic differences in percentages getting vitamin D from ≥ 2 supplement sources. All of the significant differences based on percentage estimates remained statistically significant in gender-specific models that adjusted for age and education.

Vitamin D supplementation patterns of women and men in the obese, diabetic, and hypertensive groups are shown in Table 3. Among obese adults, 40% (CI: 37.8-42.5%) of women and 51% (CI: 48.1-54.3%) of men were getting no vitamin D from dietary supplements, and only 23% (CI: 21.1-25.0%) of women and 7% (CI: 5.7-8.8%) of men were getting it from ≥ 2 sources. After adjusting for age and race-ethnicity, obese women were significantly more likely than non-obese women to get no vitamin D from supplements (OR=1.45, CI: 1.28-1.63), with no significant gender difference among men. Among diabetics, 37% (CI: 33.3-41.2%) of women and 49% (CI: 45.4-53.5%) of men were getting no vitamin D from any dietary supplement, and only 26% (CI: 22.9-30.1%) of women and 9% (CI: 7.0-11.8%) of men were getting it from ≥ 2 supplement sources. Prevalence of supplementation among hypertensives was similar to that of diabetics, likely due in part to overlap in these chronic condition groups. After adjusting for race-ethnicity, women ≥ 51 with diabetes and/or hypertension were at slightly higher risk than those who did not have either condition to be getting no vitamin D from supplements (OR=1.17, CI: 1.01-1.35), with no significant difference among men.

Among obese, diabetic, and hypertensive women, Blacks and Latinas were significantly more likely than nonHispanic Whites to get no vitamin D from dietary supplements (for Blacks and Latinas, respectively, after adjusting for age: Obese: OR=1.69, CI: 1.25-2.30 and OR=2.14, 1.62-2.84; Diabetic: OR=1.90, CI: 1.11-3.27 and OR=2.50, CI: 1.51-4.12; Hypertensive: OR=1.52, CI: 1.12-2.07 and OR=1.57, CI: 1.12-2.22). Vitamin D supplementation among Filipinas with hypertension was also significantly lower than that among nonHispanic Whites (OR=1.44, CI: 1.01-2.04). Among obese, diabetic, and hypertensive men, Latinos were significantly less likely than nonHispanic Whites to be getting vitamin D from supplements (Obese: OR=1.57, CI: 1.08-2.27; Diabetic: OR=1.73, CI: 1.04-2.90; Hypertensive: OR=1.61, CI: 1.12-2.32).

## Discussion

Due to many factors, (e.g., decrease in non-protected sun exposure, decreased consumption of vitamin D fortified foods, changing racial and age composition of the population, and increase in percentage of adults unable to use vitamin D efficiently due to advanced age, obesity, medication use, medical treatments, and health conditions), the prevalence of vitamin D insufficiency and deficiency in the population is increasing [32-34,40,45]. Based on our survey, we estimate that 40% of women aged 25–50, over 25% of women aged 51–85, and approximately 50% of men in both age groups in a relatively well-educated, insured health plan population are getting no vitamin D from any dietary supplementation. This suggests that the substantial portion of adults who are not getting adequate vitamin D from sun exposure and fortified food sources to meet the IOM’s current RDA for vitamin D are unlikely to be making up the difference with vitamin D supplements. Age- and gender-related differences in vitamin D supplementation were primarily due to differences in use of calcium with D supplements.

Similar to the NHANES-based findings of race-ethnic differences in calcium and multivitamin supplement use [50,51], we found that Black and Latina women in the 25–50 and over 50 age groups and Black and Latino men over the age of 50 were significantly less likely than nonHispanic Whites to be getting any vitamin D from dietary supplements. This is of clinical and public health concern because of the documented high prevalence of vitamin D deficiency in Black and Latino populations [33,34].

Because the results of epidemiologic studies of risks associated with vitamin D deficiency will continue to reach the public a long time before definitive recommendations based on clinical trial results are available, manufacturers of multivitamins, calcium with D supplements, and singular vitamin D, as well as producers of foods that are fortified with vitamin D, need guidance about the appropriate dosage of vitamin D to put into these types of supplements. Also, because manufacturers are likely to increase the amount of vitamin D and calcium in their multivitamin and calcium supplements based on the new IOM recommendations, people who have been taking both a daily multivitamin and calcium with D supplement may suddenly find that they are exceeding the recommended intake, although evidence suggests that dosages as high as 4,000 IU/day are not toxic [42]. In the short term, serum 25-hydroxyvitamin D (25[OH]D) samples from the most recent cycle of NHANES could be analyzed separately for males and females in different age and race-ethnic groups and for these groups by different parts of the U.S to determine extent of variation in vitamin D insufficiency and deficiency. This information about the general population could then be augmented by clinical studies to determine how high a dose of vitamin D_3_ is required to bring D-insufficient and D-deficient people up to what is considered adequate levels, resulting in more tailored DRIs for vitamin D based not only on age, but also race-ethnicity, season, and geographic location.

Our study has several limitations. First, because the overall survey response rate to this general health survey was under 50%, there is a possibility that response bias might limit the accuracy of the results. However, previous studies have found that respondents to self-administered surveys are more likely to be better educated than nonrespondents, and because numerous studies have found that health promoting behaviors are more prevalent among better educated adults, any response bias would likely result in our findings over-estimating the prevalence of vitamin D supplementation in the general population.

Another limitation of the study sample is that the numbers of Blacks, Latinos, Filipinos, and Chinese respondents used to estimate vitamin D supplementation in different race-ethnic groups were relatively small after being split across the four age-gender groups. This resulted in relatively wide confidence intervals around the estimated prevalence of supplement use. However, we obtained the same results with narrower confidence intervals in preliminary analyses using a sample of pooled 2005 and 2008 member survey respondents with nearly double the size of all the race-ethnic subgroups. The reason we decided to restrict our analyses to the 2008 survey was that the 2005 survey did not enable us to differentiate people who used calcium with D vs. calcium without D. Because the nonHispanic White subgroup was relatively large, we had sufficient power to identify several statistically significant race-ethnic differences in vitamin D supplementation.

A different type of limitation resulted from assumptions we could make about vitamin D intake from supplements. We could only estimate number of sources of vitamin D based on indication of taking multivitamins, calcium with D tablets, and singular vitamin D, not actual vitamin D intake as relates to IOM recommendations. While the normative amount of vitamin D in an adult multivitamin and calcium with D tablet in 2007–2008 was 400 IU, we do not know whether people taking calcium with D took one tablet (400 IU) or two (800 IU). Also, our estimates of the percentages of women and men getting vitamin D from ≥ 2 sources was based on the assumption that people who were taking calcium and multivitamins were doing so daily. However, when we analyzed frequency of calcium and multivitamin use data from a separate 2008 survey of members of the same health plan to test that assumption, we found that it did not hold (unpublished data). Among those who reported using both calcium and multivitamin supplements, only 45% of women and 32% of men aged 25–49, 68% of women and 55% of men aged 50–69, and 80% of women and 68% of men aged 70–84 were using both of these supplements on a daily basis, and only about 5-7% more were using both supplements at least five times a week. We also found that across both age groups, Black, Latino, and Filipino men and women users of both calcium and multivitamin supplements were significantly less likely than nonHispanic Whites to take both of them daily or at least five times a week. This suggests that our study results actually overestimates the percentages of adults who were getting vitamin D daily from at least one source, and that the extent of race-ethnic differences in vitamin D supplementation is probably underestimated.

A final limitation of our study is that we could not place vitamin D supplementation in the context of the extent to which individual respondents were in need of vitamin D from supplements based on their serum 25-hydroxyvitamin D (25[OH]D) concentration and amount of vitamin D they were getting from sun exposure and food intake. The survey did not include dietary recall data to enable analysis of potential vitamin D availability from food sources, nor did it ask about sun exposure practices. At the time of the survey, serum vitamin D tests were not routinely done, limiting the number of survey respondents for whom serum vitamin D status data would have been available. However, a recent national survey found that over 70% of nonHispanic White adults and nearly all Black and Latino adults in the U.S. were vitamin D insufficient [44]. Further, the aim of this study was to examine patterns of vitamin D intake from dietary supplements, not vitamin D intake from all sources nor the extent to which supplementation was actually desirable.

## Conclusion

Our study results suggest that a large percentage of adults do not get vitamin D from dietary supplements and that absence of supplementation is common among young women and men, older men, and people who are obese. Further, Blacks and Latinos, who are at elevated risk for vitamin D insufficiency due to darker skin pigmentation, are less likely to get vitamin D from dietary supplements than similarly aged nonHispanic Whites. Randomized clinical trials are needed to clarify the benefits and risks of vitamin D supplementation at various dosages for many of the health problems that have been linked to vitamin D deficiency by observational studies. If vitamin D is demonstrated to be efficacious in reducing chronic diseases, vitamin D supplementation may be an easy and inexpensive way to help reduce some race-ethnic health disparities and to contain burgeoning health care costs due to increasing prevalence of chronic diseases. However, until large-scale randomized clinical trials such as the VITAL study [56] clarify whether or not vitamin D intakes higher than those recommended in the recent IOM report would be beneficial, the public should at a minimum be advised about how to meet current IOM recommendations for vitamin D intake either through diet or vitamin supplementation and provision of public health screening programs for population subgroups at high-risk for vitamin D deficiency considered.

## Competing interests

The authors declare they have no competing interests.

## Authors' contributions

NPG conceived the study, study design, was the director for the health survey used for the study, and analyzed the data. NPG, MMA, and BJC collaborated on the literature review, interpretation of the results of the analyses, and the writing of the manuscript. All authors read and approved the final manuscript.

## Authors’ information

All authors are Research Scientists at Kaiser Permanente Northern California’s Division of Research in Oakland, CA.
